# Clinical factors for delayed neuropsychiatric sequelae from acute carbon monoxide poisoning: a retrospective study

**DOI:** 10.3389/fmed.2024.1333197

**Published:** 2024-02-02

**Authors:** Xin Gao, Wu Wei, Guo-Dong Yang

**Affiliations:** Department of Neurology, Jiu Jiang No. 1 People’s Hospital, Jiujiang, China

**Keywords:** carbon monoxide poisoning, delayed neuropsychiatric sequelae, exposure duration, diffusion-weighted imaging, laboratory indicators

## Abstract

**Background:**

Delayed neuropsychiatric sequelae (DNS), which seriously affect the daily lives of patients, are the most common complications of carbon monoxide (CO) poisoning. No uniform screening tool is available for identifying high-risk groups. Therefore, in this study, we aimed to explore whether conventional laboratory indicators and imaging data from primary hospitals could predict the occurrence of DNS.

**Methods:**

This retrospective observational study was conducted in a single-center primary hospital from January 1, 2021 to May 31, 2023. Participants included patients aged >18 years with acute CO poisoning. Patients with complete recovery in the acute phase were followed up by telephone and outpatient visits, and the presence of DNS was determined according to the occurrence of new neurological symptoms within 6 weeks after discharge. We obtained demographic, laboratory, and imaging data from the medical records and performed a univariate analysis. A multivariate logistic regression model was used to identify independent clinical predictors of DNS.

**Results:**

A total of 73 patients were included in the study, of whom 25 (34.2%) developed DNS. Multivariate logistic regression analysis revealed that a longer duration of CO exposure (adjusted odds ratio (AOR): 1.262, 95% confidence interval (CI): 1.069–1.490) and the presence of acute brain lesions on diffusion-weighted imaging (DWI) (AOR: 5.117, 95% CI: 1.430–18.315) were independent risk factors for DNS. Receiver operating characteristic analyses of the duration of CO exposure were performed (area under the curve (AUC): 0.825; 95% CI: 0.731–0.918) with a cut-off value of 5.5 h, and DNS was predicted with a sensitivity of 96% and a specificity of 66.7%.

**Conclusion:**

High cranial DWI signal within 24 h and duration of poisoning longer than 5.5 h are independent predictors of DNS. The predictive effects of conventional laboratory indicators require further standardized and large-sample studies.

## Introduction

Carbon monoxide (CO) is a colorless and odorless toxic gas, mostly produced by the incomplete combustion of carbon-containing compounds. It is one of the most common types of gas poisoning in daily life, with clinical symptoms varying in severity. Mild cases only present with headache, dizziness, nausea, fatigue, and listlessness, whereas severe cases can develop coma and cardiac arrest (1). Globally, the annual incidence of CO poisoning is estimated to be 137 per million, with a mortality rate of 4.6 per million (2), which may be more severe in economically underdeveloped regions and countries. CO enters the body through the lungs to form carboxyhemoglobin (COHB), which moves the oxygen-hemoglobin dissociation curve to the left, causing tissue hypoxia, especially in energy-intensive organs such as the brain and heart (3).

Neurological symptoms can manifest immediately or be delayed, predominantly in cases with COHB levels exceeding 25% (4, 5). The latter refers to the emergence of new neuropsychiatric symptoms within 6 weeks after complete recovery, which may be related to hypoxic brain injury, inflammation, free radical generation, and apoptosis. Currently, there are no unified diagnostic criteria or effective detoxification treatment (4, 6, 7). High concentrations of atmospheric oxygen are acknowledged as effective, but the risk–benefit ratio of hyperbaric oxygen therapy (HBOT) requires further evidence (8).

Because DNS can cause a considerable number of surviving patients to suffer long-term neurological and mental sequelae (4, 5), the early identification of high-risk groups is important. CO can reduce oxygen-carrying capacity, inhibit mitochondrial respiration, and produce free radicals. Once a large amount of CO is inhaled, it can damage multiple organs, such as the brain, heart, blood system, kidneys, liver, and skeletal muscle. Abnormal signals can be revealed in the corresponding cranial imaging, and the enzymes and proteins in tissues enter the blood through the damaged cell membrane. Therefore, in this study, we aimed to explore whether laboratory indicators routinely detected in primary hospitals and abnormal cranial DWI findings have value in predicting DNS.

## Materials and methods

### Study population

**Figure 1:**
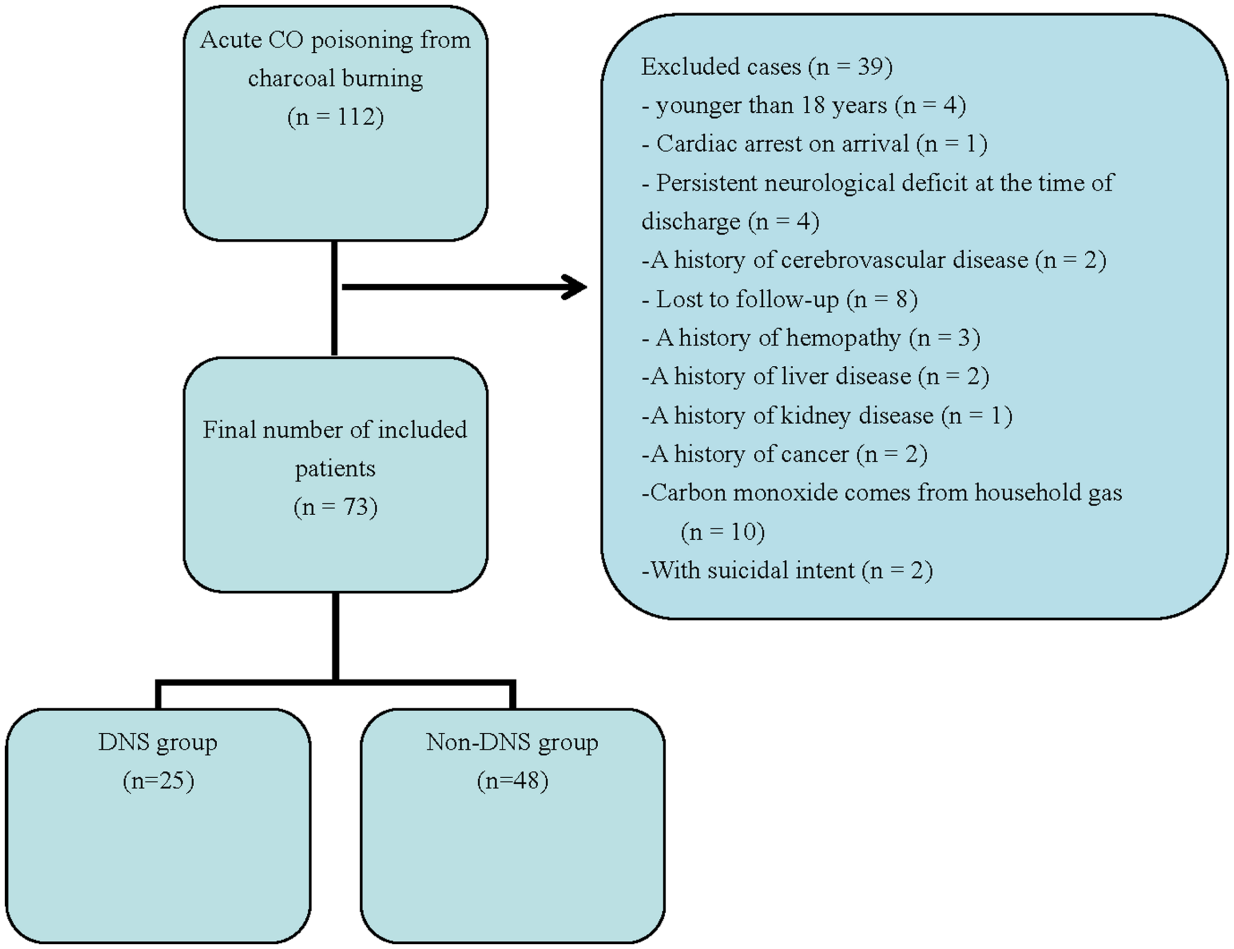
A flow chart of patient selection. DNS, delayed neuropsychiatric sequelae; CO, carbon monoxide.

The study included patients aged >18 years with CO poisoning who were admitted to the emergency department (ED) between January 2021 and May 2023. CO poisoning is defined as a history of CO exposure and the level of COHB is >5% at admission (10% for smokers) (9, 10). All enrolled patients were “unresponsive” on the AVPU (A, alert; V, responding to verbal; P, responding to pain; U, unresponsive) scale when found at the scene of poisoning (11), and blood collection was completed within 1 h of admission. Exclusion criteria included age less than 18 years, pregnancy, alcoholism, cardiac arrest prior to ED presentation, and disease history such as severe cardiovascular disease, hematological disorders, hepatitis, cirrhosis, renal insufficiency, and tumors (Figure 1). The study protocol was approved by the Ethics Committee of the Jiujiang First People’s Hospital. Written informed consent was not required due to the retrospective nature of the study.

### Study protocol

The study employed a retrospective observational analysis, focusing on CO from charcoal burning as the source. Suicide attempt cases were excluded due to potential overdoses of sedative-hypnotics and antipsychotics (12). Demographic data, comorbidities, laboratory and imaging data, and details of oxygen therapy and poisoning were collected by reviewing medical records. Cranial DWI was performed within 24 h and reviewed by two senior physicians. Neutrophil-lymphocyte ratio (NLR) was calculated based on the absolute number of neutrophils and lymphocytes in the whole blood cell count. The indications for HBOT at our center were the presence of any neurological deficits, including loss of consciousness, seizures, and a COHB level greater than 25%, regardless of the symptoms. Therefore, all patients received HBOT with specific protocols: 30 min compression, 90 min continuous maintenance (2.2 atmosphere absolute), and 30 min decompression. In addition, all patients underwent their first hyperbaric oxygen within 2 h after admission. Upon discharge, patients and their relatives were informed of the signs and symptoms of DNS, provided brochures, and were followed up by telephone and outpatient visits to categorize them into the DNS and non-DNS groups based on the presence of new neurological symptoms within 6 weeks after discharge.

### Statistical analysis

Statistical analysis was performed using SPSS 17.0 for Windows (SPSS, Chicago, IL, United States). The Kolmogorov–Smirnov test was used to determine whether the data were normally distributed. Descriptive variables are expressed as mean ± SD for data that are normally distributed and as median and interquartile ranges (25th and 75th percentiles) for variables that are not normally distributed. Descriptive variables were analyzed using the Student’s *t*-test and the Mann–Whitney *U* test. Categorical variables were analyzed using Chi-squared and Fisher’s exact tests. Variables with *p* < 0.05 in univariate logistic regression analysis were further analyzed with multivariate logistic regression analysis using the forward stepwise method. The receiver operating characteristic curve was determined to obtain the optimal cutoff value of the corresponding parameter to predict DNS. Statistical significance was set at *p* < 0.05.

## Results

A total of 112 patients with acute CO poisoning caused by charcoal burning were admitted to ED. Based on the exclusion criteria, 39 patients were excluded, and 73 patients were included in the present study (Figure 1). Since all enrolled patients were hospitalized, their demographic and clinical characteristics collected routinely in the ED could be fully traced. The baseline datas are presented in Table 1. Twenty-five patients (34.2%) developed DNS during the follow-up. There were no significant differences between the DNS and non-DNS groups in terms of sex, initial vital signs, visiting ED time, white blood cells, neutrophils, lymphocytes, glucose, lactate dehydrogenase, creatine kinase, creatinine, alanine, and aspartate transferase, NLR, comorbid diseases, or number of hyperbaric oxygen sessions. The DNS group was significantly older (45.52 ± 18.86 years vs. 61.68 ± 14.88 years, *p* < 0.001), with a longer duration of CO exposure (4.67 ± 3.94 vs. 9.24 ± 3.28 h, *p* < 0.001) and higher COHB levels (9.73 ± 2.77% vs.12.16 ± 2.12%, *p* < 0.001). DWI examinations revealed acute brain lesions in 29.16% (*n* = 14) of the patients in the non-DNS group and 80% (*n* = 20) of those in the DNS group (*p* < 0.001) (Table 1).

**Table 1:** Demographic and clinical characteristics of the study population.

	Non-DNS group (*n* = 48)	DNS group (*n* = 25)	*p*-value
Age (years)	45.52 ± 18.86	61.68 ± 14.88	0.000
Male, *n* (%)	22 (45.8%)	8 (32%)	0.254
**Initial vital signs**			
Systolic blood pressure, mmHg	119.92 ± 16.61.	122.56 ± 17.38	0.527
Diastolic blood pressure, mmHg	73.56 ± 11.15	74.16 ± 11.95	0.833
Heart rate, beats per minute	81.23 ± 12.31	77.88 ± 9.80	0.242
**Time intervals**			
Duration of CO exposure, hours	4.67 ± 3.94	9.24 ± 3.28	0.000
From exposure to visiting ED, hours	8.69 ± 5.00	11.00 ± 5.03	0.065
**Initial laboratory findings**			
White blood cell count, ×10^9^/L	11.44 ± 5.82	10.66 ± 5.30	0.578
Neutrophil count, ×10^9^/L	9.76 ± 5.79	8.79 ± 5.34	0.485
Lymphocyte count, ×10^9^/L	1.34 ± 0.61	1.44 ± 0.94	0.581
NLR	9.75 ± 8.71	8.85 ± 8.02	0.671
Glucose, mmol/L	6.45 ± 1.73	7.36 ± 2.87	0.094
Lactate dehydrogenase, ×10^9^/L	185.50 (149.75–240.75)	250.50 (95.50–829.00)	0.565
Creatine kinase, U/L	143 (79.25–440.25)	200 (159.50–276.00)	0.107
Creatinine, umol/L	60.29 ± 19.71	64.88 ± 27.88	0.709
Alanine transferase, U/L	17 (12.00–21.25)	20 (12.50–28.50)	0.272
Aspartase transferase, U/L	25 (17.25–40.75)	26 (17.50–47.50)	0.954
COHB, %	9.73 ± 2.77	12.16 ± 2.12	0.000
**Acute brain lesion on DWI, *n* (%)**	14 (29.16%)	20 (80%)	0.000
**Comorbidities**			
Hypertension, *n* (%)	4 (8.30%)	3 (12.00%)	0.614
Diabetes mellitus, *n* (%)	6 (12.50%)	4 (16.00%)	0.680
**Treatment**			
Number of HBOT sessions	9.27 ± 5.38	9.96 ± 6.79	0.637
Number of HBOT sessions ≧1 week, *n* (%)	33 (68.75%)	16 (64%)	0.773

DNS, delayed neuropsychiatric sequelae; CO, carbon monoxide; COHB, carboxyhemoglobin; NLR, neutrophil-lymphocyte ratio; DWI, diffusion-weighted imaging; ED, emergency department; HBOT, hyperbaric oxygen therapy.

**Figure 2:**
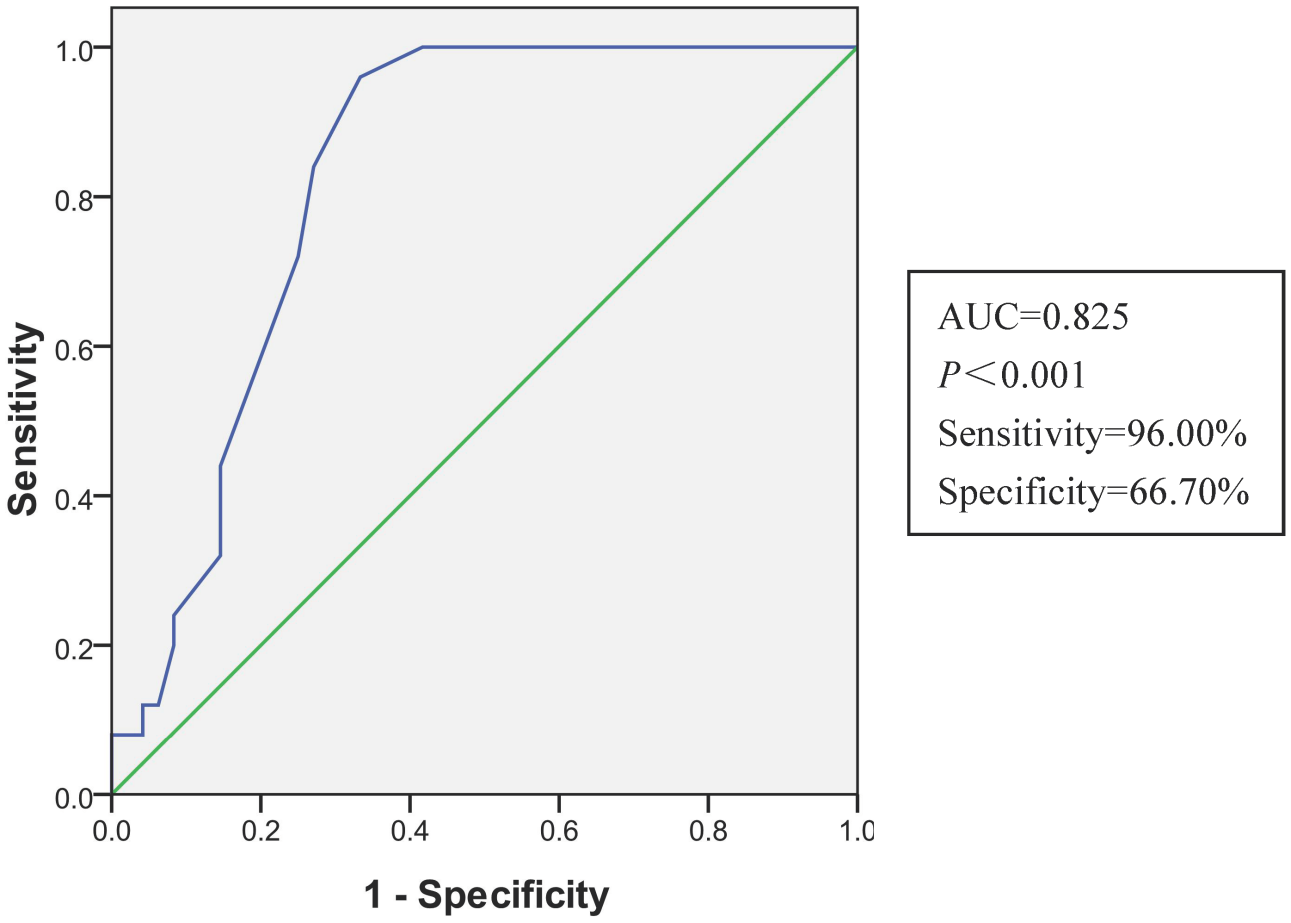
The receiver operating characteristic curve of the multivariate logistic regression model for predicting DNS. AUC, area under the curve.

Univariate analysis revealed that older age, higher COHB levels, longer duration of CO exposure, and presence of acute brain lesions on DWI were associated with DNS. The multivariate analysis found that the longer duration of CO exposure (AOR: 1.262, 95% CI: 1.069–1.490) and the presence of acute brain lesions in the DWI (AOR: 5.117, 95% CI: 1.430–18.315) were independent predictors of DNS (Table 2). The AUC of the duration of CO exposure in the receiver operating characteristic curve for the prediction of DNS was 0.825 (95%CI, 0.731–0.918). The optimal cutoff value for the duration of CO exposure was 5.5 h with a sensitivity of 96% and specificity of 66.7% (Figure 2).

**Table 2:** Predictors of delayed neurological sequelae as determined by univariate and multivariate logistic regression analysis.

Variables	Univariate analysis	Multivariate analysis
	OR (95% CI)	Value	Adjusted OR (95% CI)	Value
Age (years)	1.054 (1.021–1.089)	0.001		
Duration of CO exposure, hours	1.362 (1.158–1.602)	0.000	1.262 (1.069–1.490)	0.006
COHB, %	1.452 (1.161–1.815)	0.001		
Acute brain lesion on DWI	9.714 (3.042–31.016)	0.000	5.117 (1.430–18.315)	0.012

OR, odds ratio; CI, confidence interval; CO, carbon monoxide; COHB, carboxyhemoglobin; DWI, diffusion-weighted imaging.

## Discussion

Common clinical symptoms of DNS include memory and emotional disorders, seizures, Parkinson’s disease, movement disorders, urinary incontinence, and dysarthria. A high-signal symmetric globus pallidum is the most typical imaging sign on cranial DWI, and the incidence reported in previous studies varies from 7 to 40% (6, 13). The current study showed an incidence was 34.2%. In addition, an abnormal cranial DWI high signal intensity within 24 h and exposure duration of more than 5.5 h were found to be independent risk factors for DNS; the sensitivity and specificity of the latter were 96 and 66.7%, respectively.

Previous studies have shown that age is an independent influencing factor for DNS, and the optimal cutoff values reported were 36 and 40 years old (14, 15). Although age was not an independent risk factor in the present study, patients with DNS were significantly older than the controls. Since most permanent residents include the elderly in remote areas with relatively low educational levels, they usually lack the concept of preventing CO poisoning and do not know how to prevent poisoning (16). Therefore, public health experts are responsible for raising public awareness about the hazards and the corresponding preventive measures of CO poisoning; even CO alarms can be considered, if necessary (17).

HBOT is currently recognized as an effective treatment for acute CO poisoning, which can reverse inflammation and mitochondrial dysfunction and reduce the risk of DNS (4). However, there is no recognized consensus on the treatment; patients are generally administered one or two sessions of hyperbaric oxygen within 24 h. The incidence of DNS was not significantly different when patients were administered three sessions of hyperbaric oxygen within 24 h (25% vs. 18.8%) (5, 9). In the current study, 49 (67%) patients received HBOT once a day for at least 1 week, but the incidence of DNS seemed to be higher. A meta-analysis suggested that two sessions of HBOT did not show more than one advantage (18), but the parameters of hyperbaric oxygen in each study are inconsistent. Since hyperbaric oxygen can not only accelerate the rapid discharge of CO but also inhibit the inflammatory storm caused by poisoning (19, 20), we recommend sequential HBOT for patients who can tolerate the discomfort caused by hyperbaric oxygen and do not consider economic factors. Weaver et al. reported that hyperbaric oxygen reduces the incidence of cognitive sequelae caused by CO poisoning significantly (14). The optimal atmospheric pressure and frequency of HBOT should be the main targets in the future.

Tissue ischemia and systemic inflammation caused by CO poisoning can lead to muscle damage, and elevated creatine kinase levels can be detected in blood samples. Creatine kinase has a good predictive value for DNS; for example, Lee reported that creatinine kinase values higher than 1,603 U/L were associated with DNS, with a sensitivity and specificity of 91.7 and 88.1%, respectively (21). Another prospective cohort study showed that the cutoff creatine kinase level for predicting DNS was 175.5 U/L (9). However, the present study did not show a positive result, which is consistent with previous reports (10, 22). This difference may be explained by the various durations of poisoning and the time taken to obtain blood samples. Abnormal electrocardiography signals gradually weaken with prolonged detection time after CO poisoning, and the characteristics of muscle injury vary with the duration of hypoxia (23, 24). However, to the best of our knowledge, there have been no reports on the dynamic changes in serum creatine kinase levels over time.

Inflammation and immune-mediated injury are the main mechanisms underlying DNS development. CO can activate neutrophils through platelet–neutrophil aggregation, causing neutrophil threshing and the release of many inflammatory mediators (1, 25). As a marker of systemic inflammation and infection, NLR has a good predictive value for the improvement and deterioration of post-stroke pneumonia (26). Our study explored whether NLR had an early warning role in DNS, but the results were regrettable. Gao et al. found that the sensitivity of predicting DNS was as high as 93.88% and the specificity reached 84.43% when the NLR was greater than 8.97 (7). Moreover, NLR may also help evaluate the long-term prognosis of patients with acute CO poisoning; the greater the NLR, the worse the prognosis (27). First, Moon et al. showed that the time of blood collection affects the NLR; the neutrophil count of patients with CO poisoning showed a descending trend at admission, 6 h and 12 h, especially in patients with a poor prognosis, while the lymphocyte count did not change significantly (27). Second, we excluded factors such as hepatitis, cirrhosis, cancer, and hematological diseases that could affect the blood analysis.

The affinity of CO to hemoglobin is 250 times of oxygen, and even a short duration of exposure can lead to severe hypoxia, especially in the brain and myocardial tissues (1). Studies have shown that an exposure duration of more than 24 h is an independent risk factor for DNS. The longer the exposure duration, the higher the risk of DNS development (10, 14). However, the cutoff value of 24 h as a prognostic predictor has little guiding value in clinical practice (22). In the present study, most patients were admitted to the ED the next morning after falling asleep; the poisoning duration rarely exceeded 12 h, and if the duration of poisoning exceeded 5.5 h, the risk of developing DNS increased by approximately 1.3 times for every additional hour, which is similar to some previous studies (15, 22). The true duration of poisoning is often difficult to obtain, and patients often ignore the duration of intermittent exposure and only report the duration of continuous exposure. In addition, some patients cannot provide the true duration of exposure because of confusion and transient memory loss caused by poisoning, which is often subjectively estimated.

Cranial imaging data have been proven to predict DNS. Early studies have found that abnormal head computed tomography within 5 days after poisoning is an independent risk factor, especially for symmetrical pallidus low-density signals (28, 29). With the development of technology, DWI has become the preferred diagnostic tool for emergency physicians as it can detect cerebral hypoxic–ischemic lesions early. An observational study from South Korea showed that the sensitivity and specificity of abnormal DWI high signals in the early stage of poisoning to predict DNS were 75.2 and 90.2%, respectively (6). Our study showed that the risk of developing DNS in patients with lesions was four times higher than that in patients without lesions. In addition, another study reported that the proportion of persistent neurological damage in patients with abnormal DWI signals was significantly higher than that in healthy subjects (40% vs. 1%), which seriously affected their daily lives (30).

This study has limitations. First, this was a single-center retrospective study with a small sample size, which inevitably led to data bias. Second, some patients were followed up by telephone; among them, very mild neurological damage may have been ignored. Third, the follow-up period may have been relatively insufficient; a few patients with CO poisoning may develop DNS within 1 year (21). Multicenter studies with larger sample sizes are required to validate our results. Finally, a low Glasgow Coma Scale (GCS) score is an independent risk factor for DNS (10, 15). Although the included patients had a history of coma, we were unable to obtain homogeneous GCS scores because the prehospital emergency physicians were always fluctuant and did not receive uniform GCS scale training.

## Conclusion

Patients with an exposure duration of more than 5.5 h or lesions on cranial DWI within 24 h are a high-risk group for developing DNS, while white blood cell count, creatine kinase, and NLR have non-predictive values. However, clinical practitioners should keep in mind that a relative shorter duration of exposure or normal cranial DWI within 24 h does not indicate that patients will not develop DNS absolutely.

## Data availability statement

The original contributions presented in the study are included in the article/supplementary material, further inquiries can be directed to the corresponding author.

## Ethics statement

The studies involving humans were approved by the Ethics Committee of Jiujiang First People’s Hospital. The studies were conducted in accordance with the local legislation and institutional requirements. The human samples used in this study were acquired from a by- product of routine care or industry. Written informed consent for participation was not required from the participants or the participants’ legal guardians/next of kin in accordance with the national legislation and institutional requirements.

## Author contributions

XG: Data curation, Investigation, Writing – original draft. WW: Writing – original draft. G-DY: Writing – review & editing.
